# Triboelectric‐Based Transparent Secret Code

**DOI:** 10.1002/advs.201700881

**Published:** 2018-02-04

**Authors:** Zuqing Yuan, Xinyu Du, Nianwu Li, Yingying Yin, Ran Cao, Xiuling Zhang, Shuyu Zhao, Huidan Niu, Tao Jiang, Weihua Xu, Zhong Lin Wang, Congju Li

**Affiliations:** ^1^ Beijing Institute of Nanoenergy and Nanosystems Chinese Academy of Sciences National Center for Nanoscience and Technology (NCNST) Beijing 100083 China; ^2^ University of Chinese Academy of Sciences Beijing 100049 China; ^3^ School of Material Science and Engineering Georgia Institute of Technology Atlanta GA 30332‐0245 USA

**Keywords:** communication codes, flexible electronics, hydrophobic protection, rapid identification, self‐powered electronics, transparent electronics

## Abstract

Private and security information for personal identification requires an encrypted tool to extend communication channels between human and machine through a convenient and secure method. Here, a triboelectric‐based transparent secret code (TSC) that enables self‐powered sensing and information identification simultaneously in a rapid process method is reported. The transparent and hydrophobic TSC can be conformed to any cambered surface due to its high flexibility, which extends the application scenarios greatly. Independent of the power source, the TSC can induce obvious electric signals only by surface contact. This TSC is velocity‐dependent and capable of achieving a peak voltage of ≈4 V at a resistance load of 10 MΩ and a sliding speed of 0.1 m s^−1^, according to a 2 mm × 20 mm rectangular stripe. The fabricated TSC can maintain its performance after reciprocating rolling for about 5000 times. The applications of TSC as a self‐powered code device are demonstrated, and the ordered signals can be recognized through the height of the electric peaks, which can be further transferred into specific information by the processing program. The designed TSC has great potential in personal identification, commodity circulation, valuables management, and security defense applications.

## 1 Introduction

Smart identification has witnessed the rapid development of signal analyzing, telecommunication, human–machine interaction, and defense system.^1^, ^2^, ^3^, ^4^ With the growing attention on the mobile payment, cell phones and other intelligent devices have been commonly technological means due to their convenience, portability, and systematical organization by the software.^5^ However, excessive use of these electronics may increase the burden on the data link system and the risk of information leakage.^6^, ^7^ In daily use, there are conditions that require different identification devices, for example, in bank, shop, bus, entrance check, and security system, most of which utilize the magnetic card. But magnetic stripe, the key component, is more likely to be scratched or demagnetized.

The advent of various demands on private security has raised a significant challenge for seeking multifunctional and convenient devices that exceed the traditional limits.^8^, ^9^ These announcements promote corresponding devices to have simpler design, higher efficiency, and lower‐cost operation. However, a device combining power source and signal generation will be a good solution to the problems, especially in some independent situations.^10^, ^11^ Triboelectric nanogenerators (TENGs) are a promising field of combing energy harvester and active sensing for their simple energy conversion mechanism.^12^, ^13^, ^14^, ^15^, ^16^ Triggered by external physical motion, the TENGs are capable to detect the pressure,^17^ strain,^18^ and relative displacement.^19^ Therefore, the TENGs are widely used in different situations, for example, to sense touching,^20^ vector,^21^ vibration,^22^ velocity,^23^ and acceleration.^24^

Here, we introduce a TSC that enables both mechanical energy harvesting and information transmission by using a fluorinated ethylene propylene (FEP) film and patterned indium tin oxide (ITO) electrodes. This device is made up of three layers to form a compact, flexible, and thin piece for information identification. The injected charged particles/ions retain the local electrostatic field and make it possible for the TSC to communicate with the monitor only by surface contact.^25^ Through arranging the transparent ITO electrodes in specific patterns, the regular signals are collected by the monitor and then processed to differentiate each electric pulse. This TSC is velocity‐dependent and capable of achieving a peak voltage of ≈4 V at a resistance load of 10 MΩ at a speed of 0.1 m s^−1^, according to a 2 mm × 20 mm rectangular stripe. With conductive electrodes well protected under the film, the fabricated TSC can maintain its performance after reciprocating rolling for about 5000 times. The designed TSC has great potential in personal identification, commodity circulation, valuables management, and security defense.

## 2 Results and Discussion

**Figure 1:**
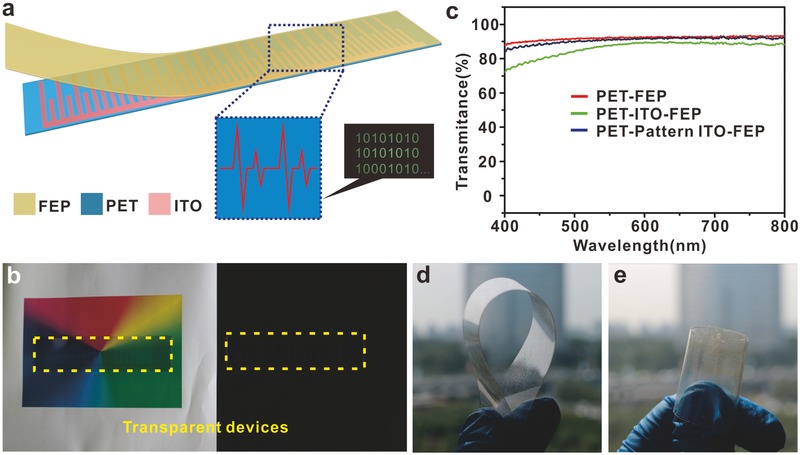
The design of the triboelectric‐based transparent secret code. a) The structure of the TSC with three simple layers. b) A TSC that is transparent in full visible color regions and easily hidden in the black background. c) Transmittance of the fabricated PET‐FEP, PET‐ITO‐FEP, and PET‐Pattern ITO‐FEP. d,e) Photographs of the flexible TSC in the bent and rolled condition.

The structure of the TSC is illustrated in **Figure** **1**a. It was like a bar code with ITO stripes of different lengths. A layer of FEP film was sealed on the top of the polyethylene terephthalate (PET)‐ITO substrate. It can be seen in Figure 1b that the fabricated device was highly transparent to all visible colors on the background of a chromatic rectangle and even invisible when it lay on a black table board, where it is framed by the yellow dashed lines. The transmittance of the device is shown in Figure 1c. It was observed that the bare PET‐FEP piece possessed the highest transmittance which is nearly 93% in visible region, a little higher than the fully covered ITO‐PET substrate sealed by FEP, and the fabricated patterned device is intermediate of which is average 90%. A little decrease of the transmittance was found from 400 to 600 nm due to the absorbance of the ITO electrode.^26^, ^27^ The whole device is long and thin, easy to bend, and convenient to take along, which possesses excellent mechanical toughness and is satisfying for daily use. We bent and rolled the as‐fabricated as shown in Figure 1d,e. It recovered easily after removing the external force.

**Figure 2:**
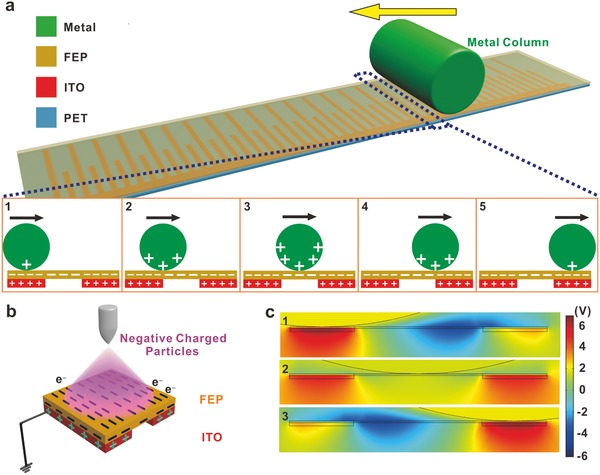
Working mechanism of the TSC. a) Schematic illustration of the working mechanism for the TSC device. b) Influenced by the local electrostatic fields, the triboelectric charges in the metal column are driven back and forth. c) Single‐polarity charged particles are injected onto the surface of the FEP film and induce the opposite charges from the ground. These charges retain at the local area. d) Theoretical stimulated results of the electrostatic fields for the sliding motion of the metal collector from the left to right side.

The working principle of the TSC is based on the single‐electrode mode triboelectric nanogenerator. The electric signals are induced by the local electrostatic field, as illustrated in **Figure** **2**a. In the initial position, the metal column contacted the surface of the FEP and gained positive charges (Figure 2b). Then the metal column rolled or slid from the left to the right side, and the changes of the induced charges are clearly illustrated (Figure 2b). It is noted that the fabricated device is pretreated by injecting single‐polarity charged particles onto the surface of FEP film, with the patterned ITO as the back electrode connected to the ground during the process, illustrated in Figure 2c. Once the metal column slides along the surface of the FEP film, the arranged ITO stripes and intervals provide nonuniform electrostatic fields, which determines the charge transfer process in the metal collector.^28^, ^29^ The simulated electrostatic field is shown in Figure 2d to better understand the potential distribution. Considering the single‐polarity charging, the electrons are attracted from the ground to the collector when the negative potential decreases by the resident positive charges in the ITO stripes, which induces the transfer current to produce the electric pulse.

**Figure 3:**
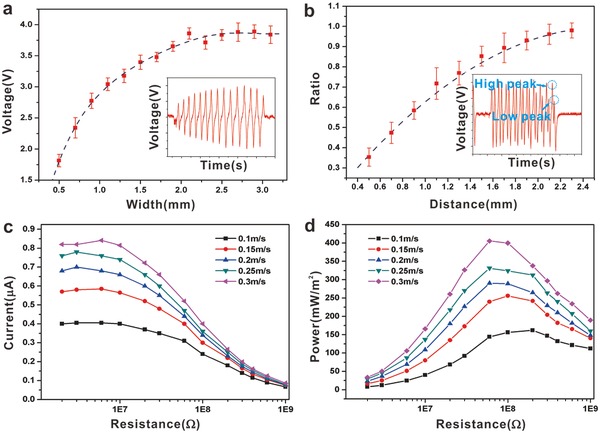
Characteristics of the ITO electrodes on the performance of the device. a) Effect of the width of the ITO stripe on the performance of the charges transfer. Inset: Illustration of the collected signals for picking up the peaks. b) The study on the distance between two adjacent electrode stripes to find out the optimum signal‐to‐noise ratio. Inset: Illustration of the collected signals for distinguishing the peaks. Output currents over different external resistance at c) different speeds and d) corresponding power density. All error bars in the figure represent standard deviation of the data and the *n* values are a) 15 and b) 13, respectively.

It can be inferred that the direct interaction between the ITO stripes and the collector has important impact on the generation of the electric signals, as shown in **Figure** **3**. Like a code bar, the induced ITO stripes are designed in rectangle, of which the long sides are vertical to the sliding direction. The elongated design increases the induced area in a short contacting time, and the length contributes to the magnitude of the electric peaks. For the same reason, the magnitude of the electric peaks also counts on the width of the stripes due to the additional charges in the limited area. We use a common iron column as the collector and push it to slide through the surface of the FEP film. A typical sample was tested carefully for the influence of the stripe width on the electric output and the results are shown in Figure 3a. It is clearly seen that the induced voltage output increases with the increasing width according to the fitted curve. Part of the collected results revealed the tendency in the inset of Figure 3a. From the results, the width beyond 2.1 mm was close to the saturation capacity, and a width of 2 mm should be an appreciate value for the following experiments. The result might depend on the curvature of the metal collector, which determined the effective induced area. In this experiment, the diameter of the metal column was 210 mm. And the optimum width of the ITO electrodes matched the charges transfer area according to the limited contact area between the collector and the code device.

The density of the information data is an important factor in practical applications. In this situation, it is determined by the amount of the ITO stripes in the limited functional area. We investigate the influence of the different intervals between two adjacent stripes. The quality factor is expressed by the ratio of high electric peak to low one of two neighboring adjacent stripes to find out the optimum distance. From 0.5 to 2.3 mm, the different ratios accord with the different intervals, shown in Figure 3b. It is noted that the mutual interference of the adjacent stripes nearly disappears when the ratio approaches 1, and the appreciate distance was a value not less than 2.1 mm.

Based on the experimental results, we obtained the optimized structural dimension for the fabricated TSC. As a self‐powered device, obvious changes in output signals were observed with larger external loads and higher velocities (Figure 3c,d). The varying curves were distinguished due to the velocities, which were displaying step‐up outputs. The maximum output power at different velocities occurred at a set of specific loads, shown in Figure 3d, and the slight displacement of the power peak was observed from the larger loads to smaller loads with increasing velocity, which was in accord with the previous reports.^30^, ^31^, ^32^

**Figure 4:**
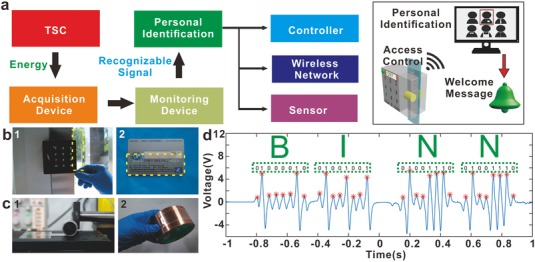
Applications of the TSC in smart identification for specific information. a) Diagram process of the TSC for intelligent identification. Inset: Illustration of a smart door access. b) Photographs showing the matching of the TSC to the door control and bank card. c) Photograph of the repetitive rolling process of the TSC and then stuck onto a hook face for electrical testing. d) The collected information from the TSC and identified to compose an expression of “BINN.”

A smart code was developed to identify the specific information, as illustrated in **Figure** **4**a. By recognizing the regularity of the signal from the TSC, the monitor can identify smartly. The message is transmitted to the controller, the wireless network, or the sensor, which can further take on complex functions. It is illustrated at the right side of Figure 4a that a smart access control system works to collect the information on the TSC and communicate with external devices such as a PC, which can process the signals to achieve personal identification and send a feedback for a welcome or refuse message. In fact, the long and narrowly strip‐shaped TSC is adaptive to a sliding‐check or roll‐to‐roll access device, which is commonly used in the door control access, ATM and transportation check‐in (Figure 4b). And the transparent and flexible characteristics of the TSC make it convenient to carry around and kept in some private situations.

The fabricated PET‐ITO‐FEP device was bearable to abuse cases. In our experiments, the TSC was easily wrapped around the metal roller back and forth (Figure 4c). And the electric signal of the device was tested after the condition of 5000 wrapping times (Figure S1, Supporting Information). Then the thin sheet was stuck onto the cambered surface of the tape reel tightly in Figure 4c. We used a homemade collector (Figure S2, Supporting Information) to sweep along the FEP film and the process was recorded by the acquisition device.

The collected information of the TSC on the positive camber is shown in Figure 4d. In this situation, the TSC was transferred into ASCII code, which was compiled by the binary system for the high electrical peak and the low electric peak standing for 1 and 0 respectively. It was observed that the signals of the device displayed orderly for distinguishing the different peaks (Figure S3, Supporting Information). The information was collected in a short time within 2 s. All the peaks were marked by the red asterisks, and considering the sequence, the results were “01000010,” “01001001,” “01001110,” and “01001110,” which represented “B,” “I,” “N,” and “N” in ASCII code, respectively. We noted that the TSC could also imply actual information by compiling the order of the electrical peaks in a special translated method.

**Figure 5:**
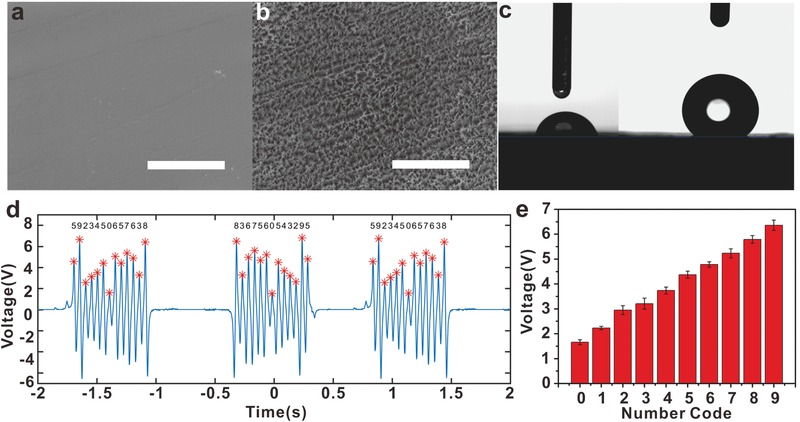
The surface modification of the TSC and code compilation based on the decimal system. a) The pre‐etching FEP film and b) the etched FEP film with nanostructured arrays. The scale bar is 5 µm. c) Photographs of the contact angles before and after ICP etching. d) The collected results of the TSC device for identifying a series of specific number code. e) The dependence of the generated signals on the length of the ITO stripes.

The TSC was further studied for improvement on the applications as an information carrier. To adapt to a variety of situations, such as overcoming water and dust contamination, the FEP film at the surface of the device was modified to be hydrophobic. The difference could be seen between the pre‐etched (**Figure** **5**a) and etched films (Figure 5b). It was inferred that the nanostructured arrays reduced the contact area of the contaminants. The nanowires could hold up the little water drop and promote the wetting angle from about 75° to 143° (Figure 5c). A rubber suction bulb was easy to blow away the liquid drop. In this way, it could be developed as a self‐clean device for its convenience to remove the water drop on the triboelectric layer, which retained the performance of the whole device.

An elaborate TSC piece was designed to carry on dense information for precise recognition. Different lengths of the stripes were substitutes for corresponding number codes. This TSC was designed with the length of the stripes ranging from 0.6 to 2.4 cm with the increment of 0.2 cm, which represented the numbers of 0–9 respectively. Through a sliding measurement process, the information of the TSC device was acquired by the collecting monitor (Figure 5d). The TSC that we used for the test was arranged in the order of “5923450657638” from one side to the other. By dividing the difference between the highest and the lowest peaks into ten detached areas evenly, we tried to find out the distribution in different regions. Similar results were obtained in the time region of (−2, −1), (0.5, 1.5). The peaks found in the regions were marked with red asterisks and the corresponding numbers, which was in the order of “5923450657638” from the left to right side. The signals in the (−0.5, 0.5) were collected when sliding in the opposite direction, and the electrical peaks agreed with the corresponding code. In the long run for the statistical regularity, the code was tested to demonstrate its reproducibility (Figure 5e, and Figure S4, Supporting Information). We believe that based on the TSC, the information compilation will have the potential applications in a more convenient, private, and safer way in daily life.

## 3 Conclusion

The paper presents a self‐powered sensor with highly recognizable information remained on a transparent and flexible thin film. The device is capable to conform to the shape or the curvature of external substrate for its excellent mechanics. It is the design of the ITO stripes of the device that works to distinguish the relative heights of the electric peaks. For the sliding or roll‐to‐roll motion from one side to the other, the length and interval of the stripes influence the output signals. This TSC is velocity‐dependent and we demonstrate their applications as a self‐powered bar code. The information was acquired by the monitor and processed to compile actual expressions. For practical applications, the cover film is modified to be hydrophobic and can be protected from the water contaminant. The designed TENG has great potential in personal identification, commodity circulation, valuables management, and security defense applications.

## 4 Experimental Section

*Fabrication of the TSC Device*: A typical PET film was chosen as the substrate. A layer of ITO was deposited onto the PET (125 µm) substrate by magneton sputtering (PVD75 Kurt J. Lesker). The designed electrodes were molded by the UV photolithography (MA6 SUSS). Then the substrate was treated with etchant to generate the desired pattern, which were connected in series by a straight conductive trace. The FEP (50 µm) film was coated with a thin layer of Cu using the magneton sputtering before it was etched by the ICP plasma (Sentech SI 500). The gases of Ar, CF_4_, and O_2_ were inlet into the ICP chamber with flow ratios of 15.0, 30.0, and 10.0 sccm, respectively. The operation chamber pressure was lower than 2 Pa, and the plasma generated power and acceleration power was 200 W and 80 W, respectively. Then the nanostructured FEP film was adhered over the PET substrate.

*Characterization for the Self‐Powered Properties*: To test the characteristics of the ITO stripes, the thin device was stuck onto a lifting platform (Zolix TSMV120‐1S). A linear motion (Linmot BF01‐37) was used to provide the mechanical motions for a metal collector (YTP LM12LUU) to slide along the FEP film. The electrical signals were collected by an oscilloscope (Tektronix DPO 2024B) and an electrometer (Keithley 6514). The different velocities of the mechanical motion were controlled by the linear motion.

*Transfer of the Electric Pulses to Information*: To reveal the code hidden in the TSC device, except for the above measurement setup, the signals were monitored by the oscilloscope and transmitted to the computer. Then the collected results were further processed by a homemade program which picked up the peaks and judged the relative difference of the peaks. According to the setup of the program, the binary or decimal system was set up to distinguish the regularity among the signals.

*Characterization of the Hydrophobic Film*: The SEM images of the etched films were carried out using a Nova NanoSEM 450 and the contact angle was measured by a XG‐CAM Contact Angle Meter.

## Conflict of Interest

The authors declare no conflict of interest.

## Supporting information

Supplementary

Supplementary

Supplementary
