# Supplementary material for: Triboelectric‐Based Transparent Secret Code
Source: Adv Sci (Weinh). 2018 Feb 4;5(4):1700881. doi: 10.1002/advs.201700881 (PMC5908373; doi:10.1002/advs.201700881)
Supplement: Supplementary file 1 — Supplementary [file ADVS-5-1700881-s002.pdf]

Copyright WILEY-VCH Verlag GmbH & Co. KGaA, 69469 Weinheim, Germany, 2018.

ADVANCED SCIENCE Open Access

# Supporting Information

for *Adv. Sci.,* DOI: 10.1002/advs.201700881

# Triboelectric-Based Transparent Secret Code

*Zuqing Yuan, Xinyu Du, Nianwu Li, Yingying Yin, Ran Cao, Xiuling Zhang, Shuyu Zhao, Huidan Niu, Tao Jiang, Weihua Xu, Zhong Lin Wang, and Congju Li**

Copyright WILEY-VCH Verlag GmbH & Co. KGaA, 69469 Weinheim, Germany, 2016.

Supporting Information

**Title**

**Triboelectric-based transparent secret code**

Zuqing Yuan, Xinyu Du, Nianwu Li, Yingying Yin, Ran Cao, Xiuling Zhang, Shuyu Zhao, Huidan Niu, Tao Jiang, Weihua Xu, Zhong Lin Wang, Congju Li*

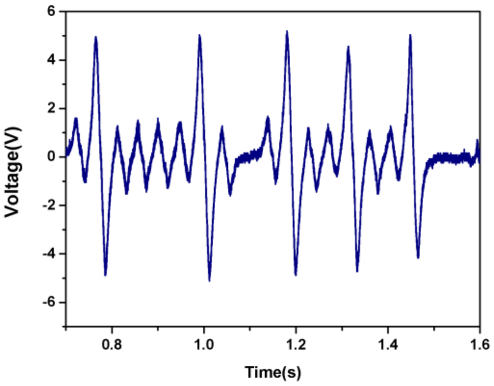


**Figure S1.** The collected signal of the TSC after 5000 times wrapping. The results were stable due to the excellent mechanical stability of the device.

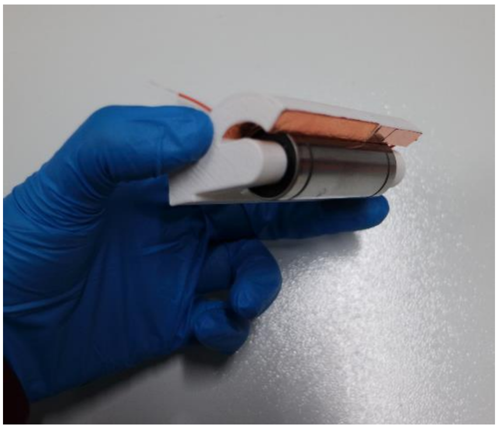

**Figure S2.** The photograph of the homemade collector by 3D print. The metal column in the middle of the device is made of stainless steel. The conductive copper foil tape was adhered to the inner side of the arc to connect the collector to the monitoring device.

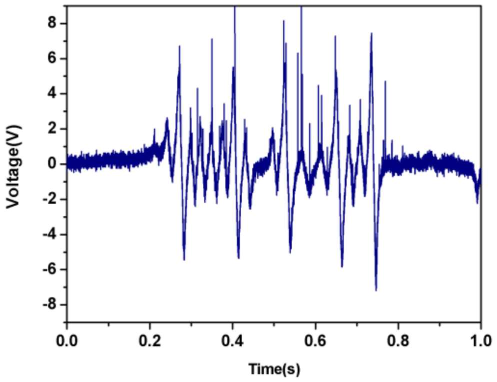


**Figure S3.** The results of the TSC on the positive camber. The high and low peak corresponded to the code number “1” and “0” respectively.

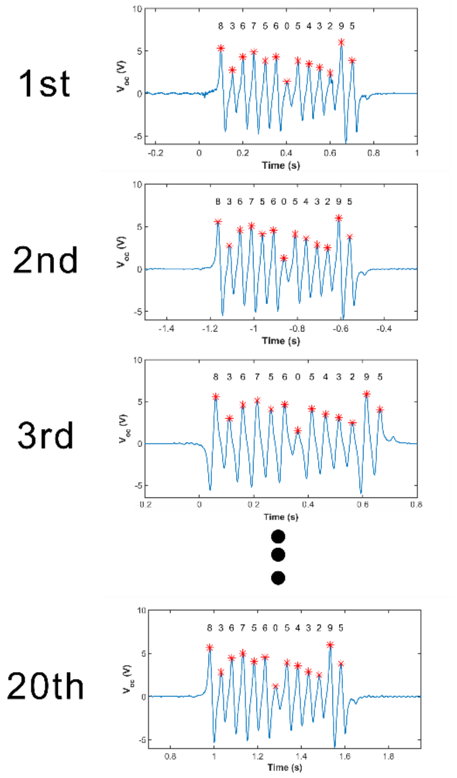


**Figure S4.** The reproducibility of the decimal code device.

Additional:

Movies S1. Hand-handling signal collected through a simple device for the TSC.

Movies S2. The Wrapping process of the flexible TSC.
